# Significant role of 1,25-dihydroxyvitamin D on serum calcium levels after total thyroidectomy: a prospective cohort study

**DOI:** 10.3389/fendo.2024.1360464

**Published:** 2024-05-13

**Authors:** Hiroyuki Yamashita, Yusuke Mori, Shinya Sato, Hisakazu Shindo, Kouichi Yoshimoto, Seigo Tachibana, Takashi Fukuda, Hiroshi Takahashi

**Affiliations:** ^1^ Department of Surgery, Yamashita Thyroid Hospital, Fukuoka, Japan; ^2^ Department of Endocrinology, Yamashita Thyroid Hospital, Fukuoka, Japan

**Keywords:** hypocalcemia, total thyroidectomy, 1, 25-dihydroxyvitamin D, Graves’ disease, thyroid cancer

## Abstract

**Introduction:**

Although active vitamin D (VD) has been used both preoperatively and postoperatively to prevent hypocalcemia risk in patients undergoing total thyroidectomy, the role of 1,25-dihydroxyvitamin D (1,25(OH)_2_D) has not been examined. This study comprehensively investigated the effects of 1,25(OH)_2_D on calcium (Ca) concentrations after total thyroidectomy.

**Methods:**

Serum Ca, parathyroid hormone (PTH), and 1,25(OH)_2_D levels were measured in 82 patients with thyroid disease before and after surgery.

**Results:**

Serum Ca, PTH, and 1,25(OH)_2_D levels decreased significantly on the morning of the first postoperative day. Notably, the decrease in 1,25(OH)_2_D concentration was significantly lower than that of PTH concentration (10.5 ± 33.4% vs. 52.1 ± 30.1%, p<0.0001), with 28% of patients showing increases in 1,25(OH)_2_D. The only factor predicting a postoperative 1,25(OH)_2_D decrease was a high preoperative 1,25(OH)_2_D concentration. Postoperative 1,25(OH)_2_D concentrations, as well as the magnitude and rate of decrease from preoperative levels, showed strong positive correlations with preoperative 1,25(OH)_2_D concentrations (p<0.0001 for all three variables) but not with PTH concentrations. These findings suggest that 1,25(OH)_2_D concentrations after thyroidectomy were more strongly dependent on preoperative concentrations than on the effect of PTH decrease and were relatively preserved, possibly preventing sudden severe postoperative hypocalcemia. A high 1,25(OH)_2_D level was the most important preoperative factor for hypocalcemia (<2 mmol/L; p<0.05) on the first postoperative day; however, only PTH decrease was statistically significant (p<0.001) when intraoperative factors were added. In the PTH >10 pg/mL group, the decrease in 1,25(OH)_2_D levels was significantly associated with postoperative hypocalcemia (p<0.05). Similarly, in the PTH levels >15 pg/mL group, a decrease in 1,25(OH)_2_D concentration was a significant factor, and the amount of PTH decrease was no longer significant.

**Conclusion:**

1,25(OH)_2_D plays an important role in preventing sudden, severe hypocalcemia due to decreased PTH levels after total thyroidectomy, whereas high preoperative 1,25(OH)_2_D levels are a significant risk factor for postoperative hypocalcemia. Optimizing preoperative protocols to adjust Ca, PTH, and 1,25(OH)_2_D levels to improve the management of patients undergoing total thyroidectomy and to prevent extreme intraoperative PTH decreases may reduce the risk of hypocalcemia.

## 1 Introduction

Hypoparathyroidism and hypocalcemia are well-documented consequences of thyroidectomy. The symptoms of hypoparathyroidism range from no symptoms to mild numbness and tingling, muscle spasms, tetany, seizures, life-threatening laryngospasm, and cardiac arrhythmias (1, 2). These symptoms not only prolong the hospital stay but also lead to increased costs for additional testing and treatment (2, 3). Various factors contribute to the risk of post-thyroidectomy hypocalcemia, including perioperative parathyroid hormone (PTH) levels, preoperative vitamin D (VD) deficiency, and potential parathyroid gland damage or removal during surgery (3). Recently, the introduction of new intraoperative modalities aimed at preserving intraoperative parathyroid function has been reported (4, 5). The literature also discusses using preoperative VD or calcium (Ca) supplementation (1, 3, 6), as well as various postoperative Ca management strategies (7, 8), to address this issue. However, evidence supporting these interventions, including those involving natural and active VD (calcitriol or alfacalcidol), remains inconclusive (9).

Our previous investigations focused on elucidating the mechanisms underlying postoperative tetany after subtotal thyroidectomy in patients with Graves’ disease (10–14). These findings attributed the tetany to secondary hyperparathyroidism (SHPT), a consequence of preoperative Ca and VD deficiency (a preoperative risk factor), and acute parathyroid dysfunction related to surgery (an intraoperative risk factor) (11). With our newly established thyroid hospital, total thyroidectomy has become the preferred surgical approach for patients with Graves’ disease and approximately half of those with thyroid cancer. Total thyroidectomy has a more significant impact on parathyroid function than other types of thyroidectomies (15, 16).

PTH is crucial in regulating Ca concentrations, both directly and indirectly, by producing 1,25-dihydroxyvitamin D (1,25(OH)_2_D). However, the difference in the half-life of PTH and 1,25(OH)_2_D (3–5 min for the former and 5–20 h for the latter) (17–19) suggests that 1,25(OH)_2_D is not completely subordinate to PTH in influencing Ca metabolism. While both PTH and 1,25(OH)_2_D are vital for managing Ca concentration after thyroidectomy, research on the role of 1,25(OH)_2_D is very limited. Consequently, our study comprehensively investigated the effects of 1,25(OH)_2_D on Ca levels after total thyroidectomy.

## 2 Materials and methods

### 2.1 Ethical considerations

The study protocol was approved by the Ethics Committee of Yamashita Thyroid Hospital (No. 2022-12). Written informed consent was obtained from all participants. All procedures were performed in accordance with the principles of the Declaration of Helsinki.

### 2.2 Study population and procedures

Eighty-two consecutive patients with Graves’ disease (n = 31), thyroid cancer (n = 42), or benign nodules (n = 9) who underwent total thyroidectomy at our hospital between June 2022 and October 2022 were included in the present study. Two patients with thyroid cancer also had Graves’ disease. One patient each with undifferentiated carcinoma, coexisting hyperparathyroidism, and completion thyroidectomy, who underwent total thyroidectomy during the same period, was excluded from the study. None of the participants had severely impaired hepatic or renal function that could have affected VD metabolism.

All surgeries were performed by five endocrine surgeons using standardized procedures. Post-surgery, the patients fasted in the following morning and received a Ca-free maintenance fluid infusion.

Serum levels of Ca, magnesium (Mg), PTH, and 1,25(OH)_2_D were measured in 82 patients before surgery, and all variables were measured again the morning after surgery. The time between thyroidectomy and postoperative blood sampling was 17.1 ± 2.0 h (range: 12.8–21.0 h). The same variables were measured 3 days and 4 weeks after surgery in patients not receiving postoperative alfacalcidol supplementation. Serum 25-hydroxyvitamin D (25(OH)D) levels were measured in all patients before surgery and 4 weeks after. Ca-related variables of all other patients were measured as needed. Ca data from the postoperative day were excluded from the analyses because two patients presented with symptoms of tetany and received intravenous Ca glucuronide before postoperative blood sampling. Postoperative hypocalcemia was defined as a corrected Ca level of < 2 mmol/L.

### 2.3 Laboratory tests

Serum levels of alkaline phosphatase (ALP; normal range, 38–113 IU/L), total Ca (2.20–2.53 mmol/L), Mg (1.6-2.6 mg/dL), albumin (34–48 g/L), and inorganic phosphate (0.87–1.49 mmol/L) were measured using routine automated procedures. The Ca level (mmol/L) was calculated using the formula: [Ca concentration (mg/dL) + 0.8 × (4 − albumin (g/dL))] × 0.250. Free thyroxine (T4, 0.9–1.7 ng/dL), free triiodothyronine (T3, 2.3–4.3 pg/mL), thyroid-stimulating hormone (TSH, 0.50–5.0 mU/L), PTH (15–65 pg/mL), anti-TSH receptor antibody (< 2 IU/L), and 25(OH)D levels were determined using an electrochemiluminescence immunoassay (COBAS 8000 e801 analyzer, Roche Diagnostics, Indianapolis, IN, USA). Serum 1, 25(OH)_2_D levels (20–60 pg/mL) were measured by radioimmunoassay (RIA) using an 125I-labelled 1,25(OH)_2_D derivative tracer and Sac-cell separation. The inter- and intra-assay coefficients of variation were 9.8 and 13%. VD deficiency was defined as a 25(OH)D concentration below 20 ng/mL, whereas VD insufficiency was described as a concentration between 20 and 30 ng/mL (20, 21).

### 2.4 Statistical analyses

Data are expressed as mean ± standard deviation. Statistical differences between the two study groups were assessed using the Mann–Whitney U test for continuous variables. Biochemical changes before and after surgery were analyzed using Wilcoxon signed-rank test. Cross-tabulated data were analyzed using Fisher’s exact probability test or Pearson’s method. Correlations were tested using Spearman’s rank correlation coefficients. Factors defining Ca and 1,25(OH)2D were tested using univariate and multivariate analyses before logistic regression analysis. Logistic regression analysis used the above significant factors to identify factors associated with hypocalcemia. All statistical analyses were performed using JMP software (version 17.0; SAS Institute Inc., Cary, NC, USA). Differences were considered statistically significant at p < 0.05.

## 3 Results

### 3.1 Overall analysis

The baseline demographic and clinical characteristics of the patients are presented in **Table 1**. The most common surgical indication for patients with Graves’ disease was the unwanted effects of antithyroid drugs (n = 14), followed by marked thyroid gland enlargement (n = 10). A preoperative cytological diagnosis of malignancy was made in 48 cases, with two additional suspected cases, all of which underwent central lymph node dissection. The suspected malignancy cases were identified as follicular carcinoma and malignant lymphoma. The preoperative diagnosis of the nine benign tumors was adenomatous goiter, and thyroid function was normal, except for TSH suppression in one case.

**Table 1:** Baseline characteristics of the study population.

Patient variables	Value (n=82)
Sex	
Male	20
Female	62
Age at surgery (years), mean (s.d.)	48.0 (16.0)
BMI (kg/m^2^), mean (range)	23.2 (16.0–39.0)
Indication for surgery	
Graves’ disease Malignancy Benign	31 42* 9
Type of lymph node dissection	
None Central lymph node dissection	40 21
Central and lateral neck node dissection (unilateral/bilateral)	21 (17/4)
Parathyroid transplantation	
None Yes (number of transplantations: 1/2/3)	24 58 (34/23/1)
Operation time (min), mean (s.d.)	130 (50)
Blood loss (g), mean (s.d.)	24 (25)
Excised thyroid gland tissue (g), mean (s.d.)	56 (46)

BMI, body mass index; s.d, standard deviation.

^*^Two patients also had Graves’ disease.

**Table 2** summarizes the pre-operative and postoperative biochemical and operative data of the 82 patients. Before surgery, serum 1,25(OH)_2_D levels showed a significant positive correlation with PTH levels (r2 = 0.155, p < 0.001) and a negative correlation with Ca levels (r2 = 0.05, p < 0.05), but not with 25(OH)D levels (r2 = 0.005, p = 0.526). Serum levels of Ca, Mg, PTH, and 1,25(OH)_2_D all significantly decreased the following day compared with their preoperative values (p < 0.001 for all four items). However, the rate of 1,25(OH)_2_D decrease was significantly lower than that of PTH (10.3% ± 32.4% vs. 52.0% ± 28.8%, p < 0.001), with a 28% (23/82) elevation.

**Table 2:** Changes in biochemical data of patients after total thyroidectomy.

Parameter	Preoperative values	Postoperative Values	Decrease amount	Decrease rate (%)	P value^*^
Serum calcium (mmol/L)	2.30 ± 0.07	2.05 ± 0.14	0.25 ± 0.15	10.9 ± 6.5	<0.001
Serum phosphate (mmol/L)	1.10 ± 0.17	1.37 ± 0.23	-0.27 ± 0.23	-26.1 ± 24.0	<0.001
Serum magnesium (mg/dL)	2.04 ± 0.15	1.76 ± 0.15	0.27 ± 0.18	13.2 ± 8.2	<0.001
PTH (pg/mL)	54.1 ± 23.3	26.5 ± 21.7	27.6 ± 19.2	52.0 ± 28.8	<0.001
1,25(OH)_2_D (pg/mL)	81.2 ± 30.6	68.0 ± 23.5	13.2 ± 28.8	10.3 ± 32.4	<0.001

1,25(OH)_2_D, 1,25-dihydroxyvitamin D; PTH, parathyroid hormone.

^*^Comparisons of serum concentrations before surgery (preoperative) and the morning after surgery (postoperative).

**Figure 1:**
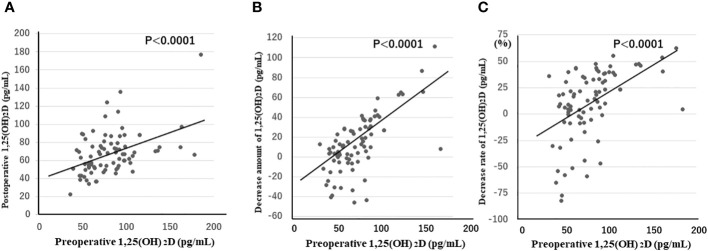
1,25(OH)_2_D concentrations **(A)**, as well as the amount **(B)** and rate **(C)** of decrease from preoperative levels in the morning after surgery, are strongly positively correlated with preoperative 1,25(OH)_2_D concentrations (p < 0.0001 for all three items). 1,25(OH)_2_D, 1,25-dihydroxyvitamin D.

The morning after surgery, 1,25(OH)_2_D concentrations, as well as the amount and rate of decrease from preoperative levels, were strongly positively correlated with preoperative 1,25(OH)_2_D concentrations (p < 0.0001 for all three items) (**Figure 1**). This was not observed for PTH concentrations. These results were consistent when patients with Graves’ disease and those with tumors were separately analyzed.

**Table 3** shows the comparison of clinical and biochemical data between the groups with decreased (n = 59) and increased (n = 23) 1,25(OH)_2_D levels. 1,25(OH)_2_D-related variables and postoperative Mg levels were significantly different; the 25(OH)D levels also differed between the two groups but without reaching statistical significance (p = 0.064).

**Table 3:** Comparison of clinical and biochemical data between patient groups with decreased and increased 1,25(OH)_2_D Levels.

Variables	Decreased levels (n=59)	Increased levels (n=23)	p value^*^
Age	46.7 ± 13.0	48.4 ± 17.2	0.668
Sex (M/F)	16/46	7/13	0.426
BMI (kg/m^2^)	22.7 ± 4.3	24.4 ± 5.1	0.145
Graves’ disease (%)	21/59 (35.6%)	12/23 (52.2%)	0.169
SHPT (PTH>65 pg/mL)	13/59 (22.0%)	4/23 (17.4%)	0.641
Preoperative laboratory findings			
Free T4 (ng/dL)	1.24 ± 0.35	1.29 ± 0.69	0.476
TSH (μU/mL)	1.49 ± 1.39	1.19 ± 0.30	0.233
PTH (pg/mL)	55.2 ± 25.6	51.5 ± 16.6	0.865
Calcium (mmol/L)	2.29 ± 0.07	2.30 ± 0.09	0.598
Phosphate (mmol/L)	1.08 ± 0.16	1.15 ± 0.17	0.116
Magnesium (mg/dL)	2.06 ± 0.15	2.00 ± 0.10	0.112
ALP (IU/L)	92.1 ± 56.6	86.4 ± 35.9	0.765
Creatinine (mg/dL)	0.65 ± 0.19	0.64 ± 0.16	0.808
1,25(OH)2D (pg/mL)	87.6 ± 32.5	64.9 ± 16.4	0.002^#^
25(OH)D (ng/mL)	15.7 ± 4.6	18.3 ± 7.7	0.345
Postoperative laboratory findings			
PTH (pg/mL)	25.6 ± 22.6	28.9 ± 19.3	0.317
PTH decrease (pg/mL)	29.6 ± 19.3	22.6 ± 18.1	0.160
Calcium (mmol/L)	2.03 ± 0.14	2.08 ± 0.16	0.403
Phosphate (mmol/L)	1.37 ± 0.24	1.34 ± 0.20	0.350
Magnesium (mg/dL)	1.74 ± 0.14	1.83 ± 0.14	0.015^#^
1,25(OH)2D (pg/mL)	62.0 ± 21.6	83.4 ± 21.6	0.001^#^
1,25(OH)2D decrease (pg/mL)	25.6 ± 22.7	-18.5 ± 15.3	0.001^#^

1,25(OH)_2_D, 1,25-dihydroxyvitamin D; 25(OH)D, 25-hydroxyvitamin D; ALP, alkaline phosphatase.

BMI, body mass index; PTH, parathyroid hormone; SHPT, secondary hyperparathyroidism; T4, thyroxine; TSH, thyroid-stimulating hormone.

Values are presented as mean ± SD or number (percentage).

^*^Comparison between the groups with decreased and increased 1,25(OH)_2_D levels.

^#^Significantly different at p<0.05.

**Table 4** summarizes the results of the univariate and multivariate analyses of the pre-operative factors defining the Ca concentration in the morning after thyroidectomy. Among the variables considered clinically important, 1,25(OH)_2_D and SHPT were significant in the univariate analysis; however, only 1,25(OH)_2_D (the higher the value, the lower the Ca concentration) was significant in the multivariate analysis (**Table 4**). Logistics regression analysis with the two significant factors defining Ca concentration showed that only 1,25(OH)_2_D levels were a predictor of hypocalcemia (< 2 mmol/L) on the morning after surgery (odds ratio: 1.22, 95% confidence interval: 1.04–1.44, p < 0.01).

**Table 4:** Univariate and multivariate analysis of preoperative factors determining the calcium concentration on the morning following the thyroidectomy.

Variable	Univariate p value	Multivariate p value
Age	0.412	
Sex	0.325	
BMI	0.580	
Graves’ disease	0.060	
TSH	0.525	
Free T4	0.189	
PTH	0.173	
SHPT (PTH >65 pg/mL)	0.035^*^	0.225
Calcium	0.155	
Phosphate	0.419	
Magnesium	0.494	
ALP	0.405	
Creatinine	0.902	
25(OH)D	0.260	
1,25(OH)_2_D	0.015^*^	0.035^*^

1,25(OH)_2_, 1,25-dihydroxyvitamin D; 25(OH)D, 25-hydroxyvitamin D; ALP, alkaline phosphatase.

BMI, body mass index; PTH, parathyroid hormone; SHPT, secondary hyperparathyroidism; T4, thyroxine; TSH, thyroid-stimulating hormone.

^*^Significant at p<0.05.

Given the strong influence of a PTH decrease on Ca concentrations and an extreme PTH decrease on the morning after surgery masking the effect of 1,25(OH)_2_D, the factors defining morning Ca concentrations (**Table 5**) and hypocalcemia (< 2mmol/L) (**Table 6**) were analyzed for all enrolled patients in addition to the groups with PTH levels >10 pg/mL or >15 pg/mL. We identified 31 cases (37.8%) of hypocalcemia on the morning after surgery in the group with all 82 patients, 19 of 61 patients (31%) in the group with PTH >10 pg/mL, and 10 of 50 patients (20%) in the group with PTH >15 pg/mL. In all cases, univariate and multivariate analyses of all factors, including intraoperative factors that might determine the Ca concentration on the morning following surgery and hypocalcemia, showed that the amount of PTH decrease was a significant parameter in multivariate analysis. In the group with PTH >10 pg/mL, the decrease in 1,25(OH)_2_D was a significant factor for Ca concentrations and hypocalcemia. Similarly, in the group with PTH levels above 15 pg/mL, the decrease in 1,25(OH)_2_D concentration was a significant factor for the two items. However, the decrease in the amount of PTH was no longer a significant factor for either.

**Table 5:** Univariate and multivariate analysis of pre- and intraoperative factors determining the calcium concentration on the morning following the thyroidectomy.

Variable	Univariate p value	Multivariate^*^ p value
Total (n=82)		
Postoperative PTH	0.008^#^	
PTH decrease amount	<0.001^#^	<0.001^#^
PTH decrease rate	<0.001^#^	
Preoperative 1,25(OH)_2_D	0.015^#^	
Postoperative 1,25(OH)_2_D	0.429	
1,25(OH)_2_D decrease amount	0.001^#^	0.119
1,25(OH)_2_D decrease rate	0.006^#^	
Postoperative magnesium	0.022^#^	0.130
Lymph node dissection	0.08	
Group with PTH >10 pg/mL (n=61)		
Postoperative PTH	0.101	
PTH decrease amount	<0.001^#^	<0.001^#^
PTH decrease rate	0.005^#^	
Preoperative 1,25(OH)_2_D	0.002^#^	
Postoperative 1,25(OH)_2_D	0.7632	
1,25(OH)_2_D decrease amount	0.001^#^	0.011^#^
1,25(OH)_2_D decrease rate	0.003^#^	
Postoperative magnesium	0.081	
Lymph node dissection	0.206	
Group with PTH >15 pg/mL (n=50)		
Postoperative PTH	0.781	
PTH decrease amount	0.004	0.176
PTH decrease rate	0.064	
Preoperative 1,25(OH)_2_D	0.001^#^	0.003^#^
Postoperative 1,25(OH)_2_D	0.844	
1,25(OH)_2_D decrease amount	<0.001^#^	
1,25(OH)_2_D decrease rate	0.003	
Postoperative magnesium	0.702	
Lymph node dissection	0.129	

1,25(OH)_2_D, 1,25-dihydroxyvitamin D; PTH, parathyroid hormone.

*To prevent the nullification of effects among relevant variables, the multivariate analysis concentrated on the reduction in the levels of PTH and 1,25(OH)2D among those variables that exhibited significance in the univariate analysis.

^#^Significant at p<0.05.

**Table 6:** Logistic analysis of pre- and intraoperative factors determining hypocalcemia (< 2mmol/L) on the morning following the thyroidectomy.

Variable	Univariate p value	Multivariate^*^ OR (95% CI), p value
Total (n=82)		
Postoperative PTH	0.002^#^	
PTH decrease amount	<0.001^#^	1.901(1.32–2.72), <0.001^#^
PTH decrease rate	<0.001^#^	
Preoperative 1,25(OH)_2_D	0.020^#^	
Postoperative 1,25(OH)_2_D	0.635	
1,25(OH)_2_D decrease amount	0.006^#^	1.16 (0.93–1.45), 0.164
1,25(OH)_2_D decrease rate	0.032^#^	
Postoperative magnesium	0.026^#^	0.74 (0.51–1.06), 0.098
Lymph node dissection	0.378	
Group with PTH >10 pg/mL (n=61)		
Postoperative PTH	0.012	
PTH decrease amount	0.001^#^	2.19 (1.30–3.67), <0.001^#^
PTH decrease rate	<0.001^#^	
Preoperative 1,25(OH)_2_D	0.013^#^	
Postoperative 1,25(OH)_2_D	0.729	
1,25(OH)_2_D decrease amount	0.005^#^	1.49 (1.01–2.01), 0.021^#^
1,25(OH)_2_D decrease rate	0.014^#^	
Postoperative magnesium	0.021^#^	0.72 (0.46–1.13), 0.150
Lymph node dissection	0.592	
Group with PTH >15 pg/mL (n=50)		
Postoperative PTH	0.342	
PTH decrease amount	0.009^#^	1.65 (0.82–3.32), 0.151
PTH decrease rate	0.012^#^	
Preoperative 1,25(OH)_2_D	0.008^#^	
Postoperative 1,25(OH)_2_D	0.692	
1,25(OH)_2_D decrease amount	0.005^#^	1.86 (1.11–3.14), 0.001^#^
1,25(OH)_2_D decrease rate	0.010^#^	
Postoperative magnesium	0.362	
Lymph node dissection	0.669	

1,25(OH)_2_D, 1,25-dihydroxyvitamin D; CI, confidence interval; OR, odds ratio (calculated per 10 pg/mL change); PTH, parathyroid hormone.

*To prevent the nullification of effects among relevant variables, the multivariate analysis concentrated on the reduction in the levels of PTH and 1,25(OH)2D among those variables that exhibited significance in the univariate analysis.

^#^Significant at p<0.05.

Regarding the VD status of the 82 patients, 66 had VD deficiency (80.5%), 14 had insufficiency (17.1%), and only 2 had sufficient VD levels (2.4%). Four weeks post-surgery, 25(OH)D concentrations significantly decreased from 16.4 ± 5.7 ng/mL to 14.9 ± 6.5 ng/mL compared with preoperative levels (p < 0.0001). The rate of 25(OH)D reduction and the pre-operative 25(OH)D concentration were negatively and significantly correlated (p < 0.05), indicating that the greater the pre-operative VD deficiency, the greater the reduction in postoperative 25(OH)D concentration.

Postoperative bleeding occurred in two patients, and recurrent nerve palsy occurred in four patients (three transient, one resected, and one nerve reconstructed due to cancer invasion). In 47 patients, alfacalcidol and Ca therapy were initiated in the morning following surgery. Of these patients, 7 (8.5%) were prescribed low doses of alfacalcidol (0.5–1.0 μg) without Ca supplementation at the last postoperative visit. Of these seven patients, three had Graves’ disease, and four had thyroid cancer. The Ca and PTH levels of these nine patients were 2.12 ± 0.03 (2.00–2.23) mmol/L and 29.6 ± 5.8 (23.5–39.5) pg/mL, respectively.

### 3.2 Sub-group analysis

**Figure 2:**
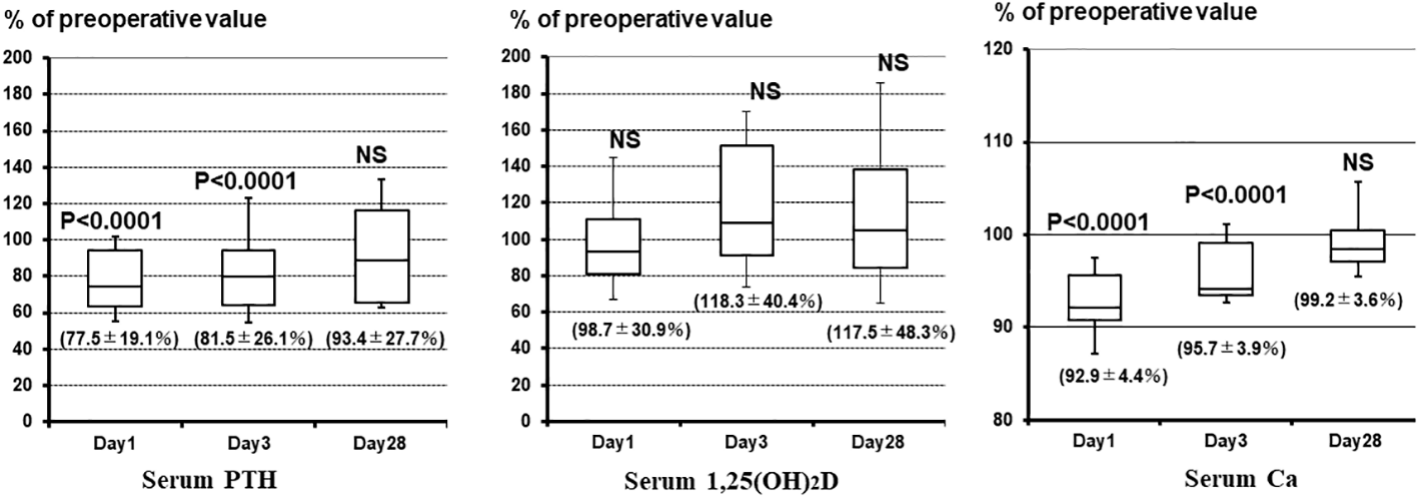
Percent changes in Ca, PTH, and 1,25(OH)_2_D concentrations from before surgery to postoperative week 4. PTH and Ca concentrations are significantly lower on the first and third days postoperatively than preoperatively; however, 1,25(OH)_2_D concentrations are not significantly different. PTH concentrations at 3 days and 4 weeks postoperatively are 10–20% lower than preoperatively, but the 1,25(OH)_2_D concentration is approximately 20% higher. 1,25(OH)_2_D, 1,25-dihydroxyvitamin D; Ca, calcium; PTH, parathyroid hormone; NS, not significant.

Changes in Ca, PTH, and 1,25(OH)_2_D levels were analyzed separately in the group (n = 28) that did not receive alfacalcidol or Ca postoperatively. **Figure 2** shows the percent changes in patients’ Ca, PTH, and 1,25(OH)_2_D levels from preoperative to postoperative week 4. PTH and Ca concentrations were significantly lower on the first and third days after surgery than preoperatively; however, 1,25(OH)_2_D concentrations were not significantly different. PTH concentrations at 3 days and 4 weeks postoperatively were 10–20% lower than those preoperatively; however, 1,25(OH)_2_D was approximately 20% higher.

Four weeks after surgery, 25(OH)D concentrations decreased significantly from the pre-operative level of 14.7 ± 4.0 ng/mL to 13.1 ± 5.7 ng/mL (p < 0.005). Similar to the analysis of all study participants, both the amount and rate of 25(OH)D reduction 4 weeks post-surgery were significantly negatively correlated with preoperative 25(OH)D concentration (p < 0.05 and p < 0.01, respectively).

## 4 Discussion

In the current study, we investigated Ca metabolism after thyroid surgery, focusing on SHPT induced by Ca and VD deficiency (11). SHPT is generally associated with normal-to-low serum Ca levels, elevated PTH levels, decreased 25(OH)D levels, and increased 1,25(OH)_2_D levels (11). The production of 1,25(OH)_2_D is achieved by 1α-hydroxylation of 25(OH)D catalyzed by the enzyme CYP27B1, which is upregulated by PTH (22). Therefore, extremely low 25(OH)D levels could result in lower 1,25(OH)_2_D levels; however, such cases are rarely seen, even in Japan, where VD deficiency is widespread (23). Similar to PTH, 1,25(OH)_2_D plays a key role in correcting hypocalcemia by promoting Ca release from the bones, renal Ca reabsorption, and intestinal Ca absorption. With a longer half-life (5–16 h) than that of PTH (3–5 min) (17–19), 1,25(OH)_2_D may have important bioprotective properties against rapid decreases in Ca levels that accompany a decrease in PTH. To the best of our knowledge, no comprehensive study has been conducted on postoperative hypocalcemia concerning 1,25(OH)_2_D levels. The lack of previous research on 1,25(OH)_2_D is probably due to its shorter half-life compared with 25(OH)D and its tendency to increase in cases of VD deficiency, making it an inadequate indicator of VD insufficiency (24, 25). In addition, its presence in serum only in trace amounts makes its measurement financially burdensome. The results of this study shed light on the complex interplay between VD metabolism, PTH regulation, and post-thyroidectomy hypocalcemia. Understanding these dynamics is critical for refining perioperative management strategies and potentially reducing the risk of hypocalcemia in patients undergoing total thyroidectomy.

**Figure 3:**
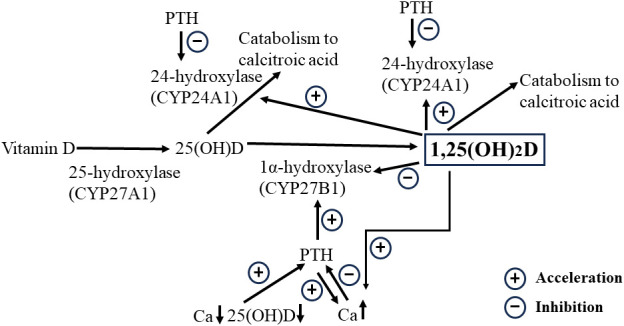
Schematic representation of the relationships among 1,25(OH)_2_D, Ca, and 25(OH)D concentrations. Increased 1,25(OH)2D due to the activation of 25(OH)D by 1α-hydroxylation facilitates Ca increases but downregulates the Ca concentration in situations where PTH depletion is likely to occur after total thyroidectomy, as it promotes catabolism through activation of the 24-hydroxylase. 1,25(OH)_2_D, 1,25-dihydroxyvitamin D; 25(OH)D, 25-hydroxyvitamin D; Ca, calcium; PTH, parathyroid hormone.

Our study revealed two important implications of 1,25(OH)_2_D after total thyroidectomy. First, 1,25(OH)_2_D significantly prevents sudden, severe postoperative hypocalcemia due to decreased PTH levels. The half-life of 1,25(OH)_2_D is considerably longer than that of PTH, suggesting that even if 1,25(OH)_2_D production is suppressed by PTH decreases, the decrease in 1,25(OH)2D is milder than that of PTH. Therefore, the Ca-raising effect of 1,25(OH)2D remained, to some extent, even when the PTH levels decreased. Furthermore, the postoperative PTH concentrations were considerably lower than preoperative levels in most cases. In contrast, the 1,25(OH)_2_D concentration decreased to a lesser degree and even increased in approximately 30% of cases. The exact mechanism is unknown; however, the activity of 1α-hydroxylase from 25(OH)D persisted to some extent even as PTH decreased, suggesting that the production of 1,25(OH)_2_D may have exceeded its catabolism in these patients. The 1,25(OH)_2_D level is thought to be primarily determined by the amount of 1,25(OH)_2_D produced from 25(OH)D by 1α-hydroxylation and its metabolism via 24-hydroxylation by the enzyme CYP24A1 (**Figure 3**) (22). Based on the results of PTH and 1,25(OH)_2_D concentrations up to 3 weeks post-surgery, the 1,25(OH)_2_D levels increased to compensate for the inadequate recovery of PTH levels. The increase in the levels of 1,25(OH)_2_D, which has a longer-lasting Ca-elevating effect than PTH does, may be a consistent physiological adaptation. Moreover, extrarenal 1,25(OH)_2_D production may be involved, but the mechanism needs further investigation (26).

Second, a high preoperative 1,25(OH)_2_D level was a significant risk factor for postoperative hypocalcemia. Furthermore, higher levels of 1,25(OH)_2_D increase the formation of 1,24,25-trihydroxyvitamin D through catabolism by 24-hydroxylase (22, 27, 28). Given that the higher the pre-operative 1,25(OH)_2_D concentration, the higher the amount and rate of decrease in 1,25(OH)_2_D concentration, the postoperative reduction in 1,25(OH)_2_D levels is likely attributed to the metabolic effects of high preoperative 1,25(OH)2D concentrations rather than to the reduced 1,25(OH)_2_D production due to postoperative PTH decrease. This finding prompts the reevaluation of active preoperative VD supplementation protocols because it could induce CYP24A1, increasing 1,25(OH)_2_D catabolism, which may lead to postoperative hypocalcemia. Optimizing preoperative Ca, PTH, and 1,25(OH)2D levels may be crucial for reducing the incidence of hypocalcemia after thyroidectomy.

The risk of hypocalcemia after total thyroidectomy was also studied by grouping patients according to their PTH levels. The clinical significance of 1,25(OH)_2_D cannot be properly determined at PTH-deficient concentrations. The results showed that a decrease in 1,25(OH)_2_D concentration in the group with PTH levels above 15 pg/mL was the only significant factor for next-morning Ca concentration and hypocalcemia. Preoperative control of risk factors and improvement of surgical techniques to prevent intraoperative PTH decreases represent the two main approaches to preventing hypocalcemia after total thyroidectomy. Such decreases can be controlled to some extent by advances in surgical techniques and adjunctive technologies. PTH is not a significant risk factor for hypocalcemia when the PTH level is 15 pg/mL or higher on the day after surgery (20% of patients in this group had hypocalcemia in the present results), suggesting that improving the 1,25(OH)_2_D levels before surgery will reduce the incidence of hypocalcemia.

The findings of this study provide new insights into various hypocalcemic etiologies. First, we discuss the “hungry bone syndrome” in postoperative patients with Graves’ disease and hyperparathyroidism who have hyperdynamic bone disease. It is often observed that prescribing high doses of active VD and Ca in these patients does not result in the expected increase in serum Ca (29, 30). It is thought that the high preoperative PTH and 1,25(OH)_2_D levels lead to a hypercatabolic state of 1,25(OH)_2_D, which is further aggravated by the postoperative PTH decrease (PTH is known to inhibit 24-hydroxylase activity) (**Figure 3**) (31), while the Ca-elevating effect of 1,25(OH)_2_D is severely suppressed. Prescribing high doses of active VD for such a condition may further activate 1,25(OH)_2_D catabolism-promoting 24-hydroxylase; therefore, it would be appropriate to provide intravenous Ca infusion therapy during this period and then wait for PTH recovery and a decrease in bone hypermetabolism. Second, in clinical practice, the timing of hypocalcemia onset varies among patients, even when PTH levels are similarly reduced, and 1,25(OH)_2_D may also be involved. In other words, although a decrease in PTH concentration lowers the 1,25(OH)_2_D levels, the rate of decline depends not only on the PTH decrease but also on the pre-operative 1,25(OH)_2_D concentration. A slower rate of decline in 1,25(OH)_2_D may delay the onset of hypocalcemia. Finally, some differences in active VD requirements for postoperative hypocalcemia at similar levels of PTH reduction may be partly explained by the rate of 1,25(OH)_2_D metabolism. It has been noted that VD deficiency increases 1,25(OH)_2_D metabolism (22, 27, 28), and thus, the prescription requirements would be expected to be higher than those in VD-sufficient patients. As discussed below, many patients require an active VD prescription even if their postoperative PTH is normal. Many of these patients may not only have low levels of 25(OH)D, the substrate for 1alpha-hydroxylase, but may also have hypermetabolism of 1,25(OH)_2_D.

The American Thyroid Association guidelines strongly recommend preoperative assessment of Ca and 25(OH)D levels, repletion if necessary, or prophylactic administration (32). Low serum 25(OH)D concentration is the most important diagnostic marker of VD deficiency (33, 34). In the present study, VD deficiency was also found to be common in Japanese patients, with only 2 of 82 patients meeting the criteria. The mean 25(OH)D concentration in this study was 16.4 ng/mL, which is the same as the 16.4 ng/mL reported in our previous study in 2000 (12). 1,25(OH)_2_D values are elevated in VD deficiency, but there was no negative correlation between serum 25(OH)D and 1,25(OH)_2_D values in the present study. Since 1,25OHD has a significant positive correlation with PTH, we speculate that in Japan, where 25(OH)D and Ca deficiency is prevalent, 1,25(OH)_2_D may rely more on PTH than on 25(OH)D as a substrate. The Japanese Ministry of Health, Labour and Welfare recommends a daily Ca intake of at least 700 mg for men and 650 mg for women, but in a preliminary survey of our inpatients, less than 10% of patients met this recommendation (data not shown). Japanese food is known to have a good balance of nutrients, and Ca is efficiently absorbed, but Japanese are gradually moving away from Japanese food, and there are few foods fortified with Ca and VD. Additionally, Japanese people, especially women, tend to avoid ultraviolet rays for cosmetic reasons, and few people take VD supplements.

The 25(OH)D concentration at 4 weeks postoperatively was significantly lower than that preoperatively. It is also noteworthy that the rate of decline was higher in patients with lower 25(OH)D. Factors that might be related to 25(OH)D concentrations after surgery, such as postoperative 1,25(OH)_2_D concentrations and the presence or absence of active VD and/or medications, were examined; however, no significant associations were found between these variables and the decrease in 25(OH)D. As the patients were 4 weeks postoperatively, lower UV production due to seasonal variations in UV radiation was unlikely, and it is possible that they were less active outdoors after surgery. Seven patients were taking alfacalcidol at the last postoperative visit, although their PTH levels were normal, and it is possible that 25(OH)D, the substrate for 1α-hydroxylation, is not sufficient in these patients. Under the Japanese National Health Insurance system, 25(OH)D measurement is not allowed only for suspected VD deficiency, and natural-type VD is not covered by insurance. Due to these problems, it is necessary to educate patients about VD deficiency measures and lobby the government for insurance coverage of 25(OH)D testing and natural-type VD.

The results of this study can be used to discuss preventive measures against hypocalcemia in patients undergoing total thyroidectomy. The adequacy of VD and Ca differs with the country and region and must be addressed according to these circumstances (35). In general, serum 25(OH)D is lower at higher latitudes and with darker skin types, and its prevalence ranges from 24% in the United States to 90% in the Middle East (35). In Japan, the frequency of SHPT caused by VD and Ca deficiency is high, and it is imperative to improve the SHPT status. Ideally, only natural VD and adequate Ca supplementation are desired; however, improvements are expected to take time. The amount and duration of VD and Ca needed to improve SHPT status should be considered. Active VD (calcitriol or alfacalcidol) and Ca would raise serum Ca to the upper limit of the normal range in a short period and lower PTH levels, both of which benefit postoperative hypocalcemia. However, preoperative active VD administration may adversely affect postoperative Ca concentrations by increasing the preoperative 1,25(OH)_2_D level and 24-hydroxylase activity (**Figure 3**). The duration of 24-hydroxylase induction by operational VD administration, which promotes catabolism, remains unknown. Therefore, the appropriate dose and duration of active VD and Ca administration should be considered.

Although this study provides valuable insights into the effects of 1,25(OH)_2_D on Ca metabolism in patients with Graves’ disease and thyroid tumors undergoing total thyroidectomy, it has some limitations. The frequency of SHPT due to inadequate VD and Ca intake is high in Japan, and its sufficiency varies in different countries and regions; therefore, the results of this study may not apply to all countries. Nevertheless, our findings will be helpful in countries and regions where SHPT is less frequent. In such areas, preoperative identification of patients with SHPT and targeting these patients for treatment to improve SHPT would be more efficient than treating all patients uniformly. Patients with Graves’ disease differ significantly from those with other thyroid diseases in terms of preoperative thyroid hormone status and the bone- and Ca-related effects of thyroid hormones and thyroid-stimulating antibodies (3, 10, 11). Most surgical indications for our patients with tumors were peritracheal or lateral neck malignancies requiring lymph node dissection, which tend to have decreased postoperative PTH levels compared with Graves’ disease. The greater the number of cases and the greater the variability in PTH decrease after thyroidectomy, the better the understanding of the role of 1,25(OH)_2_D. Furthermore, the most important finding of this study was that 1,25(OH)_2_D levels on the morning of the first postoperative day were dependent on preoperative 1,25(OH)_2_D levels, which was confirmed when patients with Graves’ disease and tumors were analyzed separately.

In conclusion, we demonstrated the important role of 1,25(OH)_2_D in preventing sudden, severe hypocalcemia due to decreased PTH levels after total thyroidectomy. High preoperative 1,25(OH)_2_D levels were significant risk factors for hypocalcemia. This study provides a basis for reviewing current clinical practice and developing more effective strategies for managing patients undergoing total thyroidectomy, with the aim of reducing the burden of hypocalcemia and improving patient outcomes. Further studies are warranted to validate these findings and refine perioperative protocols to improve patient care.

## Data availability statement

The datasets presented in this article are not readily available because The datasets generated or analyzed during the current study are not publicly available because of the institution’s policy but are available from the corresponding author upon reasonable request. Requests to access the datasets should be directed to Hiroyuki Yamashita, yamaftc@kojosen.com.

## Ethics statement

The studies involving humans were approved by Ethics Committee of Yamashita Thyroid Hospital. The studies were conducted in accordance with the local legislation and institutional requirements. The participants provided their written informed consent to participate in this study.

## Author contributions

HY: Conceptualization, Formal analysis, Writing – original draft, Writing – review & editing, Data curation, Methodology. YM: Conceptualization, Data curation, Writing – review & editing, Formal analysis, Validation, Project administration. SS: Conceptualization, Data curation, Writing – review & editing, Validation. HS: Data curation, Writing – review & editing, Investigation, Validation. KY: Data curation, Writing – review & editing, Investigation. ST: Data curation, Writing – review & editing. TF: Data curation, Writing – review & editing. HT: Writing – review & editing, Conceptualization, Supervision.
